# A novel EEG-based major depressive disorder detection framework with two-stage feature selection

**DOI:** 10.1186/s12911-022-01956-w

**Published:** 2022-08-06

**Authors:** Yujie Li, Yingshan Shen, Xiaomao Fan, Xingxian Huang, Haibo Yu, Gansen Zhao, Wenjun Ma

**Affiliations:** 1 School of Computer Science, South China Normal University, Guangzhou, China; 2 College of Big Data and Internet, Shenzhen Technology University, Shenzhen, China; 3 Department of Acupuncture and Moxibustion, Shenzhen Traditional Chinese Medicine Hospital, Shenzhen, China

**Keywords:** Depression detection, Two-stage feature selection, EEG

## Abstract

**Background:**

Major depressive disorder (MDD) is a common mental illness, characterized by persistent depression, sadness, despair, etc., troubling people’s daily life and work seriously.

**Methods:**

In this work, we present a novel automatic MDD detection framework based on EEG signals. First of all, we derive highly MDD-correlated features, calculating the ratio of extracted features from EEG signals at frequency bands between $\beta$ and $\alpha$. Then, a two-stage feature selection method named PAR is presented with the sequential combination of Pearson correlation coefficient (PCC) and recursive feature elimination (RFE), where the advantages lie in minimizing the feature searching space. Finally, we employ widely used machine learning methods of support vector machine (SVM), logistic regression (LR), and linear regression (LNR) for MDD detection with the merit of feature interpretability.

**Results:**

Experiment results show that our proposed MDD detection framework achieves competitive results. The accuracy and $F_{1}$ score are up to 0.9895 and 0.9846, respectively. Meanwhile, the regression determination coefficient $R^2$ for MDD severity assessment is up to 0.9479. Compared with existing MDD detection methods with the best accuracy of 0.9840 and $F_1$ score of 0.97, our proposed framework achieves the state-of-the-art MDD detection performance.

**Conclusions:**

Development of this MDD detection framework can be potentially deployed into a medical system to aid physicians to screen out MDD patients.

## Introduction

Major depressive disorder (MDD) is a debilitating disease characterized by at least one discrete depressive episode lasting no less than 2 weeks, which involves clear-cut changes in mood, interests and pleasure, as well as changes in cognition and vegetative symptoms [1]. It is one of the three major diseases throughout the world, and its prevalence is still on the rise [2]. MDD may affect a patient’s life severely when it attacks. Patients suffering from MDD are often accompanied by symptoms such as anhedonia, appetite and physical changes, poor sleep quality, slow thinking, loss of willpower activity, easy fatigue, excessive self-blame, and so on. In clinical practice, the most used methods for MDD diagnosis are mainly dependent on professional depression rating scales like the 17-item Hamilton rating scale (HAMD-17) [3]. However, the rating scale assessment conducted requires a trained physician available, which is difficult for community and township hospitals to provide such kind of MDD detection services. Herewith, developing an automatic MDD detection method has great significance.

In recent years, EEG signals are widely being employed in brain function research and clinical applications, such as Brain-Computer Interfacing (BCI) [4], emotion classification [5], epilepsy [6, 7], and dementia [8]. Not limited to these, researchers also utilize EEG signals as a tool to build an MDD detection model. EEG signals are formed by the summation of postsynaptic potentials occurring simultaneously in many neurons in the brain, divided into $\delta$, $\theta$, $\alpha$, $\beta$, and $\gamma$ bands [9]. In addition, it contains rich human physiological information, which can be used to discover the features connected to depressive disorders. Although the existing methods [10–13] have been achieved promising results in MDD detection, these methods have not sufficiently been solved the following challenges: (1) How to find highly MDD-correlated features with medical interpretations. (2) How to reduce the feature searching space on high-dimensional data.

Many researchers attempted to generate MDD-related features from EEG signals to solve the first challenge. Specifically, Hosseinifard et al. [9] extracted features directly from EEG band power and nonlinear features of detrended fluctuation analysis (DFA), higuchi fractal, correlation dimension and lyapunov exponent from EEG signals utilizing signal processing. Acharya et al. [14] presented an MDD-related feature named depression diagnosis index (DDI) through the combination of different nonlinear features, including fractal dimension, largest Lyapunov exponent, and sample entropy. Mahato et al. [15] derived EEG-based linear features of band power, interhemispheric asymmetry, and nonlinear features of relative wavelet energy (RWE) and wavelet entropy (WE) and fused these features together for depression detection. In general, most of the MDD-related features presented in their work were derived through signal processing or mathematical analysis from EEG signals. However, these features were not proven to be directly correlated to MDD in prior medical research and MDD detection performance. Instead, their correlation needs to be deduced backward according to the classification results of MDD detection models. Therefore, there is no sufficient evidence to show that these features are highly correlated with MDD, and how to introduce the prior medical research knowledge when extracting the highly correlated features of MDD remains to be solved.

For the second challenge, Mohammadi et al. [16] employed the designed feature selection method of genetic algorithm (GA) on the features extracted by linear discriminant analysis (LDA), aiming to achieve the optimal MDD detection result. However, GA is a randomized searching method, which is unstable and still has the potential risk of falling into the optimal local solution. Wajid et al. [17] utilized a rank-based feature selection method by assigning weight and a rank-ordered to an EEG feature matrix. Then, the features were ranked-ordered according to the scores calculated by the area under the curve (AUC). And finally, the parts with the highest scores were selected. Afterwards, Wajid et al. [18] further improved their feature selection method. They first ranked the extracted features in descending order according to receiver operating characteristics (ROC), then splited the features into subgroups to train the classifiers, and ultimately selected the subgroup whose features could make the classifier achieve the optimal result. However, these methods select features based on AUC and ROC metrics, which would overemphasize the importance of ranking, and thus ignore the information of the recall rate. In addition, these wrap-based feature selection methods have high feature searching space. Especially for high-dimensional data, the computational complexity is quite high.

To solve the above challenges, in this study, we propose a novel EEG-based framework for MDD detection and severity assessment with a two-stage feature selection. First of all, 92 participants at Shenzhen Traditional Chinese Medicine Hospital with a signed consent form were recruited to collect EEG signals with assessing MDD severity with the HAMD-17 scale by a physician. Then, highly MDD-correlated features are derived based on the ratio of extracted features from EEG signals at frequency bands between $\beta$ and $\alpha$. Subsequently, a two-stage feature selection method called PAR with the combination of Pearson correlation coefficient (PCC) and recursive feature elimination (RFE) is presented with the advantage of minimizing the feature searching space. Finally, we employ widely used machine learning methods of LR, SVM, and linear regression (LNR) for MDD detection and MDD severity assessment with the merit of well feature interpretability. Experiment results show that our proposed framework can obtain promising results, where the best $F_{1}$ score and regression determination coefficient $R^{2}$ are 0.9846 and 0.9479, respectively. Differed in the multi-modal method [19] requiring multiple inputs such as facial expressions, heart rate, and posture, the input of our framework is based on EEG signals, which is much more convenient for community/township hospitals to implement. The main contributions of this study can be summarized as follows:

1. We derive highly MDD-correlated features called $\beta /\alpha$ ratio features calculated by the ratio of extracted features from EEG signals at frequency bands between $\beta$ and $\alpha$, which can greatly improve the MDD detection performance and strong medical interpretation.
2. To further minimize the feature searching space, we present a two-stage feature selection method with the combination of PCC and RFE.
3. We utilize widely used machine learning methods of LR, SVM, and LNR for MDD detection and MDD severity assessment with well interpretability.
4. Extensive experiment results show that the proposed MDD detection framework has superiority to the state-of-the-art methods with a big margin.

The remained contents of this paper are organized as follows. In “Related work” section , the literature review of MDD detection is presented. In “Methods” section, the details of the proposed framework are briefly introduced, including the data collection, data preprocessing, two-stage feature selection, and MDD detection model. In “Results” section, MDD detection experiment results are presented. In “Discussion” section, hyper-parameters, limitations and future work are discussed. Finally, this study is summarized in “Conclusion” section.

## Related work

In recent years, physiological signals, including EEG [6], ECG [20, 21] and EMG [22], have been widely used in disease detection. With the development of machine learning (ML) technology, many researchers have made outstanding contributions to automatic MDD detection using ML. Various deep learning methods [23, 24] have been proposed. Specifically, Sharma et al. [25] presented an EEG-based network for depression screening with CNN and LSTM. Seal et al. [26] proposed a designed deep CNN framework for detecting depression using EEG signals. Although these deep learning methods can achieve high accuracy, their selected features lack interpretability, which is a big defect in medical applications where interpretability is highly valued. Although traditional machine learning methods are slightly inferior to deep learning methods in the accuracy of MDD detection, MDD-related features for both machine use and human analysis are selected through feature engineering combined with prior knowledge of humans, and these features are accessible to humans to understand.

There have been several machine learning methods employing feature engineering to detect MDD. Akbari et al. and Mohammadi and Moradi [27, 28] utilized the reconstructed phase space (RPS) of EEG signals and geometrical features for training the SVM classifier and KNN classifier for depression recognition. Jiang et al. [29] extracted features from spatial information of EEG signals to detect MDD. Sadiq et al. [30] introduced a unified algorithm to classify neural diseases from two distinct EEG domains. However, most of their proposed models have only used mathematical or signal processing methods to extract EEG-related features, which are less correlated with MDD, resulting in poor performance of MDD detection. Therefore, how to extract MDD-related features from EEG signals for better detection remains to be solved.

Some previous researchers [24, 31, 32] attempt to improve the performance of ML methods by extracting MDD-related features from EEG signals. Ciarleglio et al. [31] derived features from EEG by a brain function measure named Frontal power asymmetry (FA). Avots et al. [32] tried different linear and nonlinear EEG features, including relative band power, spectral asymmetry index, Higuchi fractal dimension, and Lempel–Ziv complexity. Bailey et al. [33] extracted features by exploring the resting EEG $\theta$ connectivity or $\alpha$ power to the predictors of response to rTMS treatment. Although the aforementioned MDD detection methods could extract MDD-related features, they generally employ one-stage feature selection, which tends to search local optimal selected features from high dimensional feature search space, whose MDD detection performance still exists an improvement margin.

## Methods

**Fig. 1:**
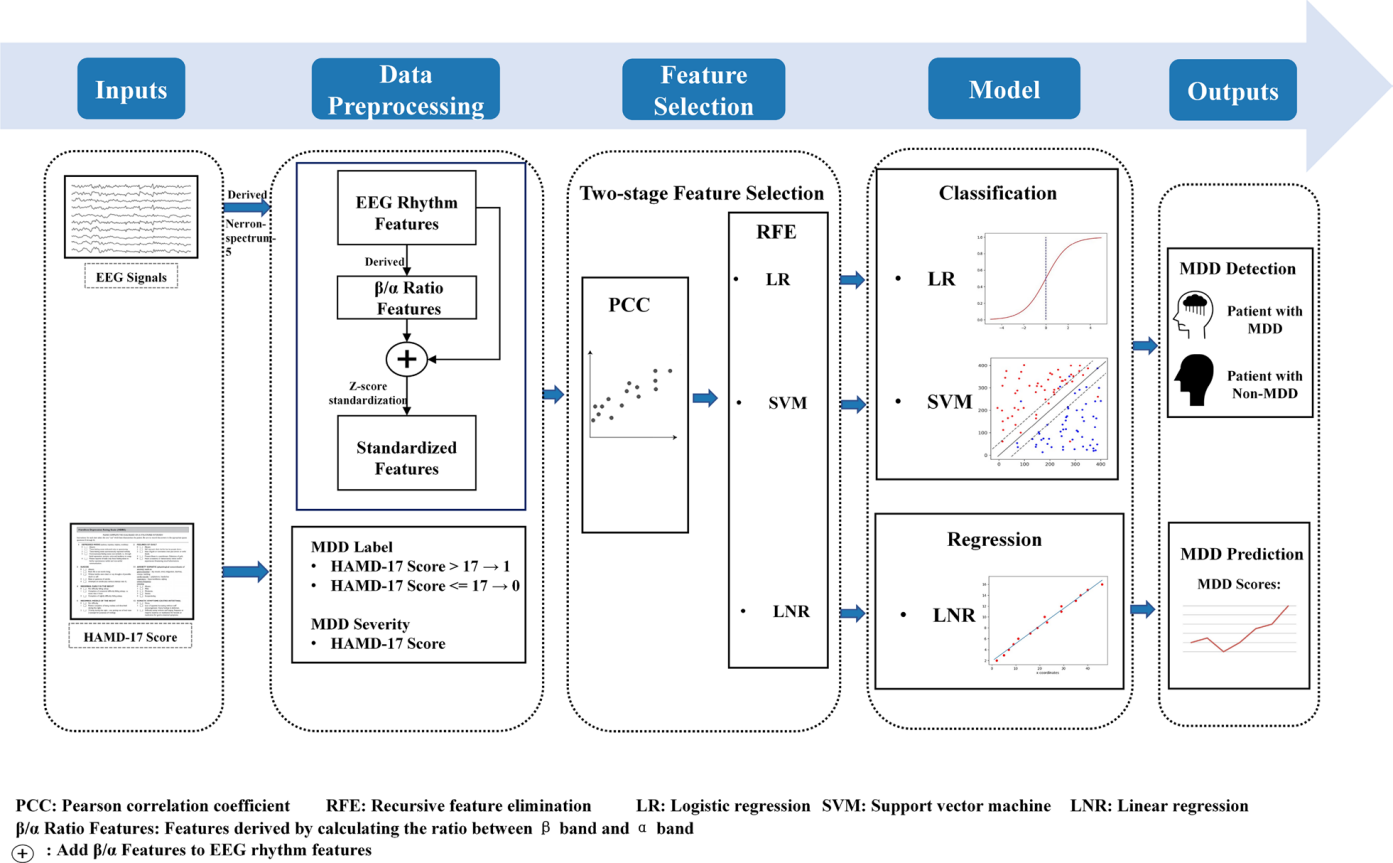
Proposed framework for MDD detection and severity prediction: Firstly, in the input module of the framework, the raw EEG signals are derived by Nerron-Spectrum-5 to obtain the EEG rhythm features, and the subjects are diagnosed and scored by a physician to get the HAMD-17 score. Then, in the data processing module, the $\beta /\alpha$ features are extracted from the EEG rhythm features and are Z-score standardized together with the rhythm features to obtain standardized features. Subjects with HAMD-17 scores greater than 17 are labeled as MDD, and those with HAMD-17 scores less than or equal to 17 are labeled as non-MDD. Moreover, the HAMD-17 score directly served as an indicator of MDD severity assessment. Then, in the feature selection module, PCC carries out the first stage feature selection on standardized features, and RFE carries out the second stage feature selection on reserved features. Finally, LR and SVM are used as classification models to classify subjects into MDD and non-MDD. LNR is used as the regression model to assess the severity of MDD, and the HAMD-17 score predicted by LNR is used as the severity indicator of MDD

The proposed framework mainly consists of data preprocessing, feature selection, MDD detection and severity prediction. Figure 1 shows the whole pipeline of our framework.

### Data collection

92 participants with depression lasting more than two weeks, who were conscious, and did not get aphasia or mental retardation, were recruited from the Shenzhen Traditional Chinese Hospital and signed informed consent forms (IRB No. 2017-8) to collect EEG signals and corresponding HAMD-17 scores. A 10–20 system of electrode placement was placed on the scalp of the participants, and smooth EEG signals were acquired with the Nerron-spectrum-5 EEG device for 30 seconds under the condition of eyes closed. The Nerron-spectrum-5 EEG device has a total of 19 channel leads, which are FP1-A1, FP2-A2, F3-A1, F4-A2, FZ-A2, C3-A1, C4-A2, CZ-A1, P3-A1, P4-A2, PZ-A2, O1-A1, O2-A2, F7-A1, F8-A2, T3-A1, T4-A2, T5-A1, and T6-A2. Each channel-lead can automatically obtain EEG-related features based on the information of rhythm waveform amplitude indexes and rhythm indexes. Both indexes possess frequency bands of $\delta$, $\theta$, $\alpha$, and $\beta$. Therefore, 152 EEG-related features are obtained from each participant. With additional demographic features of gender and age, a total of 154 features are derived for subsequent processing and analysis.

### Data preprocessing

Clinically, frequency band $\beta$ in EEG is associated with an alert or excited state of mind and frequency band $\alpha$ is more dominant in a relaxed state, both of them are associated with brain inactivation. Previous studies [34, 35] were also shown that the $\beta /\alpha$ ratio features are potentially correlated with MDD, which are derived with EEG frequency bands of $\alpha$ and $\beta$. Thus, in this work, the $\beta /\alpha$ ratio features are derived by calculating the ratio between frequency bands of $\beta$ and $\alpha$ on each channel-lead in terms of EEG waveform amplitude indexes and rhythm index information, which are defined to be:

1 $$\begin{aligned} F_{A}&= \frac{F_{A-\beta }}{F_{A-\alpha }} \end{aligned}$$

2 $$\begin{aligned} F_{R}&= \frac{F_{R-\beta }}{F_{R-\alpha }} \end{aligned}$$

where features of $F_{A}$ and $F_{R}$ are the $\beta /\alpha$ ratio features, derived from extracted features of EEG waveform amplitude indexes and rhythm indexes respectively. $F_{A-\beta }$ and $F_{A-\alpha }$ are features from EEG waveform amplitude indexes on frequency bands of $\beta$ and $\alpha$ in EEG signals, respectively. $F_{R-\beta }$ and $F_{R-\alpha }$ are features from EEG rhythm indexes on $\beta$ and $\alpha$ frequency bands, respectively. After calculation, a total of 38 $\beta /\alpha$ ratio features are derived, which are combined with the extracted feature set of EEG waveform amplitude index and rhythm index. Totally, there are 192 features obtained for feature selection.

In the MDD detection task, participants are divided into two groups according to the HADM-17 score to reflect the severity of MDD. Those with a HADM-17 score greater than 17 are defined as MDD, labeled as 1; those with a HADM-17 score no more than 17 are defined as mild depression or without depression, labeled as 0. In the MDD severity assessment task, the real HAMD-17 scores are compared with the predicted ones in the LNR-related model. As reported in [10, 17, 18], z-score standardization is widely used in EEG-based MDD detection task. Therefore, in order to minimize the impact of different feature scales, we standardize the data on each feature using z-score standardization for numerical data, making different features to be in a uniform distribution. Since the obtained EEG features might not be centered and unequally distributed, z-score standardization is more suitable than min-max normalization, which can be affected by possible outliers in the dataset. Z-score standardization is defined to be:

3 $$\begin{aligned} z=\frac{x-\mu }{\sigma } \end{aligned}$$

where ${\mu }$ is the mean value of a feature and ${\sigma }$ is the variance of it. In addition, for the non-numerical feature of gender, male is marked to be 1 and female is to be 0.

### Two-stage feature selection

After data preprocessing, directly using the obtained high-dimensional features as input usually cause poor performance in the classification task and the regression task. In addition, in feature selection, if the features with the highest correlation with MDD can be selected while reducing the feature searching space, the model can achieve the best results. Therefore, selecting an appropriate algorithm to conduct feature selection is essential before building a specified model. Typical single-stage feature selection methods usually use filters [36] to set thresholds to remove low correlation features or use wrappers [37] to optimize the given learner directly. Specifically, the filters have the advantages of simplicity and efficiency, and the wrappers have the advantages of high accuracy in the task of ML. However, during the selection process, the former can not obtain adequate information from the learner, resulting in poor performance of the learner, while the latter requires multiple iterations in all the input features to train the learner, and many low-correlation features are also iterated, resulting in high computational cost. Therefore, it is appropriate to employ the wrappers model on the features retained by the filters, which can effectively minimize the feature searching space.

To take advantage of the low resource consumption of the filter and the high performance of the wrapper simultaneously, in this work, we present a two-stage feature selection method named PAR with the combination of filter method PCC [38], and wrapper method RFE [39]. Specifically, we first define the PCC threshold as $\tau$, and RFE selected feature number as $\zeta$, then the PCC method, which is a simple tool to calculate the correlation coefficient between the EEG features and HAMD-17 scores, is selected for the first stage of feature selection. We employ PCC to calculate the absolute correlation coefficient (values between 0 and 1) between the features and the labels (the values of labels are 1 for MDD, and 0 for non-MDD) as PCC values, and then sort the corresponding features according to the PCC values in reverse order. Then, the features with PCC values less than $\tau$ are removed, and the features with PCC values greater than $\tau$ will be retained for the second stage of feature selection. The second-stage feature selection method is employed with the RFE, which has the advantage of utilizing the supervision information to select optimal features. In the second stage, RFE first recursively eliminates the features selected by PCC and deletes the number of features to the specified number $\zeta$. Then it builds the model based on the remaining features and calculates the average score ($F_{1}$ score in MDD detection and $R^2$ in MDD severity assessment) through five-fold cross-validation. Finally, the feature combination that contributes the most to the prediction results and its corresponding hyper-parameters $\tau$ and $\zeta$ are saved. The final selected features and hyper-parameters $\tau$ and $\zeta$ are determined by the grid search technique described in the Algorithm 1. In general, in grid search, we determine the hyper-parameters $\tau$ and $\zeta$ that can make the model perform best in five-fold cross-validation through a two loop. The former hyper-parameter is the threshold value used by PCC to select features, and the latter is the number of features used by RFE to select features. Employing RFE feature selection on the features selected by PCC first can improve computing efficiency. Finally, the features obtained through two-stage feature selection are not only MDD high related, but also can be better utilized by the learners (LR, SVM, and LNR). To better describe our feature selection algorithm PAR, we write a pseudo-code which is described in detail in Algorithm 1.
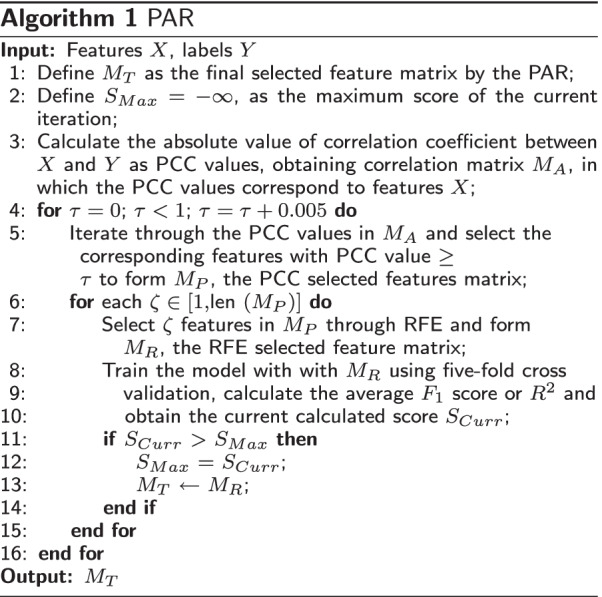


In Algorithm 1, we first define the final selected feature matrix as $M_{T}$, and the maximum measurement score of the current iteration as $S_{Max}$. Then, we calculate each PCC value between input EEG features *X* and their corresponding label *Y* (MDD or non-MDD) and combine the PCC values to form the PCC matrix $M_{A}$. And then, in a two loop, the hyperparameter $\tau$ of the outer loop is tuned from 0 to 1 with a step of 0.01, controlling the correlation of the retained features filtered by PCC in the first stage of feature selection, and the selected features are saved as matrix $M_{P}$. The hyperparameter $\zeta$ of the inner loop is tuned from 192 to 1 with a step of 1, controlling the number of features selected by RFE in the second stage of feature selection, and the selected features are saved as matrix $M_{R}$. The retained EEG features are then used to train the model (SVM and LR in MDD detection, and LNR in MDD severity assessment), and the average score $S_{Curr}$ ($F_{1}$ score in MDD detection, and $R^{2}$ in MDD severity assessment) is calculated using 5-fold cross-validation. Next, the calculated $S_{Curr}$ is compared with $S_{Max}$, and if the value of $S_{Curr}$ is greater than the value of $S_{Max}$, it will be assigned to $S_{Max}$, and the features of $M_{R}$ will be assigned to $M_{T}$. The next iteration of the loop is then carried out. Finally, the EEG features that make the model perform best are saved as matrix $M_{T}$ for MDD detection or severity assessment, and feature analysis.

### MDD detection

MDD detection task can be essentially transformed into a binary classification problem, by classifying patients with MDD and non-MDD. After two-stage feature selection, the widely used machine learning methods LR and SVM are used for MDD detection in this work.

LR [40] is a widely used classification method, which utilizes the Sigmoid function as a posteriori probability distribution to identify patients with MDD or not. Meanwhile, LR is with merits of less computation, interpretability, and easy implementation, the equation [41] is defined to be:

4 $$\begin{aligned} P(y=1|x)&= \frac{exp(\theta ^{T}x)}{1 + exp(\theta ^{T}x)} \end{aligned}$$

5 $$\begin{aligned} P(y=0|x)&= \frac{1}{1 + exp(\theta ^{T}x)} \end{aligned}$$

where $\theta$ is the weight importance of the LR model, $y=1$ denotes patients with MDD as well as $y=0$ denotes patients not with MDD.

SVM [42] is a binary classification method originally, and its decision boundary is the maximum-margin hyperplane, the equation [43] is defined to be:

6 $$\begin{aligned} w^{T}x+b=0 \end{aligned}$$

where *w* is weight importance and *b* is bias. The SVM uses the hyperplane to discriminate patients into MDD or not. Owing to the merit of human-interpretability, the SVM used in this work is with a linear kernel function [43], which is defined to be:

7 $$\begin{aligned} k(x_{i}, x_{j}) = x_{i}^{T} x_{j} + c \end{aligned}$$

where $x_{i}$ and $x_{j}$ are vectors of different EEG rhythm signal samples, *c* is an optional constant.

### MDD severity assessment

The MDD severity assessment can be transformed into a linear regression problem, and the HAMD-17 score is used as an indicator of depression severity, because the more severe the depression, the higher the corresponding HAMD-17 score will be. Therefore, we need to use the features obtained through two-stage feature selection to fit the measurement metric, to realize the assessment of the depression severity in patients. LNR [44] has a rapid modeling ability and is very effective for input data with a small sample size. In addition, it has a good explanation for each variable, which has been widely used in the medical field. LNR uses the minimum square function called linear regression equation [45] to model the relationship between one or more independent variables and dependent variables, which is defined as follows:

8 $$\begin{aligned} \begin{aligned} {\hat{y}}&=\beta ^{T} x + d \end{aligned} \end{aligned}$$

where ${\beta }$ is the parameter of the model, *x* is the vector of the EEG rhythm signal sample, and *d* is an optional constant.

### MDD detection performance metrics

Typical classification metrics, including sensitivity (*Sen*), specificity (*Spec*), precision (*Prec*), recall (*Rec*), accuracy (*Acc*), and $F_{1}$ score are used for each class. They are defined as:

9 $$\begin{aligned} { Sen}&=\frac{T P}{T P+F N} \end{aligned}$$

10 $$\begin{aligned} { Spec }&=\frac{T N}{T N+F P} \end{aligned}$$

11 $$\begin{aligned} {Acc}&= \frac{TP + TN}{T P+F P + FN + TN} \end{aligned}$$

12 $$\begin{aligned} { Prec }&=\frac{TP}{T P+F P} \end{aligned}$$

13 $$\begin{aligned} { Rec }&=\frac{T P}{T P+F N} \end{aligned}$$

14 $$\begin{aligned} {F_{1}}&=\frac{2 *({ Prec } * { Rec })}{ { Prec }+ { Rec }} \end{aligned}$$

where TP refers to the number of actual MDD patients classified to be MDD, FP refers to the number of actual non-MDD patients classified to be MDD, FN refers to the number of actual MDD patients classified to be non-MDD, and TN refers to the number of actual non-MDD patients classified to be non-MDD.

### MDD severity assessment performance metrics

Typical regression metrics, including mean absolute error ($L_{MAE}$) [46], mean squared error ($L_{MSE}$) [47], determine coefficient $R^2$ [48] are employed to evaluate the performance of LNR. They are defined as:

15 $$\begin{aligned}&{L_{MAE}}=\frac{1}{n} \sum _{i=1}^{n}\left| {y}_{i}-{\hat{y}}_{i}\right| \end{aligned}$$

16 $$\begin{aligned}&{L_{MSE}}=\frac{1}{n} \sum _{i=1}^{n}\left( y_{i}-{\hat{y}}_{i}\right) ^{2} \end{aligned}$$

17 $$\begin{aligned}&{R^{2}}=1-\frac{\sum _{i=1}^{n}({\hat{y}}_{i} - y_{i})^{2}}{\sum _{i=1}^{n}(y_{i} - {\bar{y}})^{2}} \end{aligned}$$

where ${y}_{i}$ is the actual HAMD-17 score for each individual, ${\hat{y}}_{i}$ is the corresponding predicted score, and ${\bar{y}}$ is the mean value of all HAMD-17 scores.

## Results

### Experimental environment

The proposed framework is implemented with python 3.6.11 and scikit-learn 0.21.3. All the experiments are performed on a laptop equipped with an AMD Ryzen 7 4800U CPU and 16 GB memory.

### MDD detection performance

In this work, two widely used machine learning methods of LR and SVM, with the merit of good feature interpretability, are employed to build the MDD detection model. Furthermore, to evaluate the MDD detection model generality, the five-fold cross-validation technique is used. With the hyperparameters of LR and SVM keeping default, experiment results show that the LR with derived $\beta /\alpha$ features (LR-DF for short) achieved 0.5677, 0.6182, 0.5984, and 0.5050 in terms of sensitivity, specificity, accuracy, and $F_{1}$ score, respectively. For the SVM with derived $\beta /\alpha$ features (SVM-DF for short), its MDD detection performance is comparable to the LR-DF, which achieved a sensitivity of 0.5333, specificity of 0.6894, accuracy of 0.6318, and $F_{1}$ score of 0.5144. With the two-stage feature selection, the MDD detection performance of both LR-DF and SVM-DF are improved greatly. The SVM-DF with the two-stage feature selection obtained the best MDD detection performance, which are 0.9714, 1.0000, 0.9895, and 0.9846 in terms of sensitivity, specificity, accuracy, and $F_{1}$ score, respectively. It means that the SVM-DF with two-stage feature selection can be potentially deployed into a medical system to provide an automatic MDD detection service.

### MDD severity assessment performance

To further help physicians to obtain patients’ MDD severity, the specified HAMD-17 score should be provided. In this work, the LNR is used for MDD severity assessment with default hyperparameters in the machine learning framework scikit-learn. As shown in Table 1, the LNR with the derived $\beta /\alpha$ features (LNR-DF for short) achieves better performance for MDD severity assessment than that without the derived $\beta /\alpha$ features, which means that the derived features can boost the performance of MDD severity assessment as well as MDD detection. Since the input parameter matrix required by LNR fitting is a nonsingular matrix, and we only have 92 data, there are 154 features of LNR and 192 features of LNR-DF without feature selection, which leads to the result that input rank is higher than the number of experimental data. Therefore, the $L_{MAE}$, $L_{MSE}$, and $R^{2}$ value of the LNR and LNR-DF model without feature selection cannot be calculated. Compared with the LNR, the LNR-DF achieved better MDD severity assessment performance, whose determine coefficient is 0.0927 with PCC selection, and 0.8962 with RFE selection. In PCC feature selection, the features selected by LNR are $F_{A-\delta }^{FZ-A2}$, $F_{A-\delta }^{CZ-A1}$, and Gender, and the features selected by LNR-DF are $F_{R-\theta }^{FP2-A2}$, $F_{A-\delta }^{F3-A1}$, $F_{A-\delta }^{FZ-A2}$, $F_{A-\delta }^{CZ-A1}$, $F_{A-\delta }^{O2-A2}$, $F_{A-\delta }^{T4-A2}$, $F_{R-\beta /\alpha }^{FP2-A2}$, and Gender. These features generally have a strong correlation with each other, resulting in that the learning region of the model can only be limited to a small range, and cannot make good use of other features with less close correlation, eventually lead to poor performance. Furthermore, with the two-stage feature selection, MDD severity assessment performance of both LNR and LNR-DF is improved a lot. It can be observed that the LNR-DF has much superiority over the LNR on the task of MDD severity assessment, whose determine coefficient can be up to 0.9479. It means that LNR-DF with two-stage feature selection can regress the ground truth of the HAMD-17 score very well and can be a reference for physicians to assess the patients’ MDD severity.

**Table 1:** MDD severity assessment performance

Feature selection	Model	*L*_*MAE*_	*L*_*MSE*_	*R*^2^
PCC	LNR	3.6917	21.6649	0.0644
	LNR-DF	3.5198	20.9566	0.0927
RFE	LNR	1.5306	3.4942	0.8474
	LNR-DF	1.2450	2.3995	0.8962
PCC and RFE	LNR	1.2799	2.7388	0.8812
	LNR-DF	0.9123	1.2012	0.9479

## Discussion

### MDD detection performance analysis

To verify the effectiveness of the derived $\beta /\alpha$ features and two-stage feature selection, extensive experiments are performed in this study. As shown in Table 2, both LR-DF and SVM-DF, with the derived $\beta /\alpha$ features, achieve better MDD detection performance than those methods of LR and SVM without the derived $\beta /\alpha$ features. It demonstrates that the derived $\beta /\alpha$ features have high correlations with the MDD detection task. Meanwhile, it can also be observed that MDD detection methods of LR, SVM, LR-DF and, SVM-DF raise their performance greatly no matter single-stage or two-stage feature selection. More importantly, the MDD detection methods with the RFE achieve better performance than those with the PCC, which is the reason why we choose the RFE as the core feature selection in the presented two-stage feature selection method.

Specifically, as shown in Table 2, the derived $\beta /\alpha$ features can help MDD detection methods to improve their performance more or less. Take MDD detection models of LR, SVM, LR-DF, and SVM-DF with the two-stage feature selection for example, the LR and SVM which are not with the derived $\beta /\alpha$ features achieved $F_{1}$ scores of 0.7803 and 0.9581, respectively. The LR-DF and SVM-DF which are with the derived $\beta /\alpha$ features obtained $F_{1}$ scores of 0.8713 and 0.9846, which outperform the LR and SVM with margins of 11.66% and 2.77%, respectively. What’s more, it can be observed that MDD detection methods of LR, SVM, LR-DF, and SVM-DF improve their performance a lot with feature selection, especially with the two-stage feature selection. For example, the SVM-DF with single-stage feature selection achieved $F_{1}$ scores of 0.5766 and 0.9667 when the PCC and RFE are employed respectively, which obviously outperform the SVM-DF without feature selection. Regarding the presented two-stage feature selection, the MDD detection performance of SVM-DF increases significantly to the SVM-DF without feature selection or with single-stage feature selection, which is up to $F_{1}$ score of 0.9846. It means that the presented two-stage feature selection is effective in the task of MDD detection.

**Table 2:** MDD detection performance of the proposed framework

Feature selection	Model	*Sen*	*Prec*	*Spec*	*Acc*	*F*_1_
–	LR	0.4476	0.3776	0.6167	0.5557	0.4005
	SVM	0.4524	0.4809	0.7061	0.6139	0.4510
	LR-DF	0.5667	0.4728	0.6182	0.5984	0.5050
	SVM-DF	0.5333	0.5000	0.6894	0.6318	0.5144
PCC	LR	0.5619	0.5061	0.6530	0.6207	0.5235
	SVM	0.5381	0.5444	0.7591	0.6782	0.5306
	LR-DF	0.6238	0.6083	0.7394	0.6961	0.5988
	SVM-DF	0.5619	0.6442	0.7727	0.6969	0.5766
RFE	LR	0.7381	0.8571	0.9106	0.8466	0.7803
	SVM	0.9143	0.9667	0.9833	0.9579	0.9385
	LR-DF	0.8524	0.8298	0.8955	0.8800	0.8386
	SVM-DF	0.9429	1.0000	1.0000	0.9789	0.9667
PCC and RFE	LR	0.7381	0.8571	0.9106	0.8466	0.7803
	SVM	0.9714	0.9464	0.9652	0.9678	0.9581
	LR-DF	0.8238	0.9417	0.9636	0.9127	0.8713
	SVM-DF	0.9714	1.0000	1.0000	0.9895	0.9846

### Comparison of MDD detection performance

In addition, the proposed framework is compared with state-of-the-art methods. Table 3 shows the performance comparison of MDD detection between the proposed framework and cutting-edge methods published in recent years. Here, we have to point out that the test datasets used in this work and cutting-edge methods are different because the datasets in the medical domain are often private and difficult to be accessed publicly. Even so, it still makes significant sense when cutting-edge methods are compared on a general level. As shown in Table 3, the best model SVM-DF of the proposed framework has obvious superiority to cutting-edge methods in terms of sensitivity, accuracy, and $F_{1}$ score. Regarding the specificity, the SVM-DF achieves 0.98, which is still comparable to the best specificity of 1.0 reported by [17].

**Table 3:** Performance comparison with cutting-edge MDD detection methods

Method	*Sen*	*Spec*	*Acc*	*F*_1_
[9]	–	–	0.9000	–
[10]	0.9490	0.8090	0.8790	–
[11]	0.9444	–	0.8912	–
[12]	–	–	0.8500	–
[17]	0.9666	1.0000	0.9840	–
[18]	0.9990	0.9500	0.9800	0.9700
[13]	–	–	0.7927	–
[49]	–	–	0.8833	–
SVM-DF	0.9714	1.0000	0.9895	0.9846

### MDD severity assessment performance analysis

**Fig. 2:**
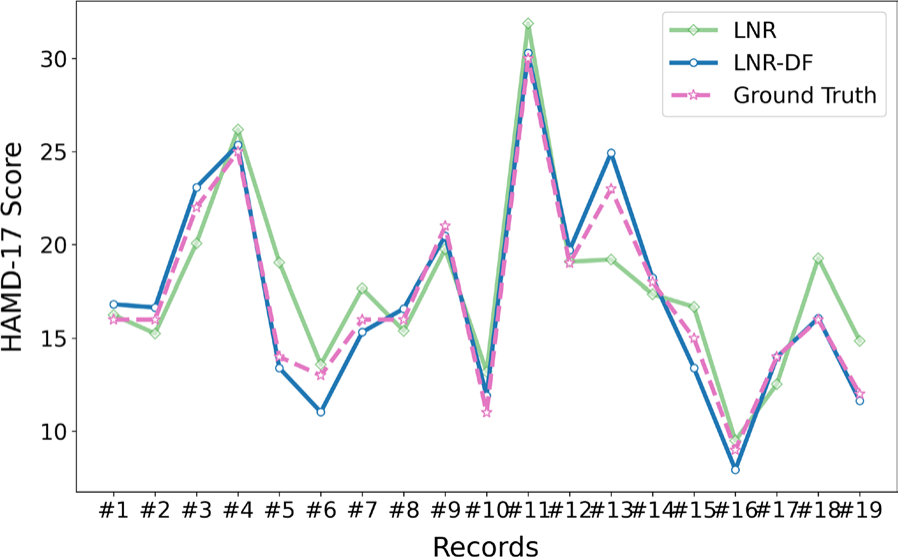
MDD severity assessment performance on random selected 10 records

To further verify the effectiveness of the MDD severity assessment model, 19 records from individuals are randomly selected for validation. As shown in Fig. 2, it is the MDD severity fitting performance chart with the two-stage feature selection, where the x-axis and y-axis refer to the record and HAMD-17 score, respectively. Most of the cases, the LNR and LNR-DF can well predict the MDD severity represented by the HAMD-17 score. However, there exists a big difference in MDD severity assessment performance on the records 18 and 19. Throughout the whole Fig. 2, the results of LNR-DF are closer to the ground truth than those of the LNR. It demonstrates that the presented two-stage feature selection has positive effects on the task of MDD severity assessment and the MDD severity assessment model can improve its performance with the derived $\beta /\alpha$ features.

**Fig. 3:**
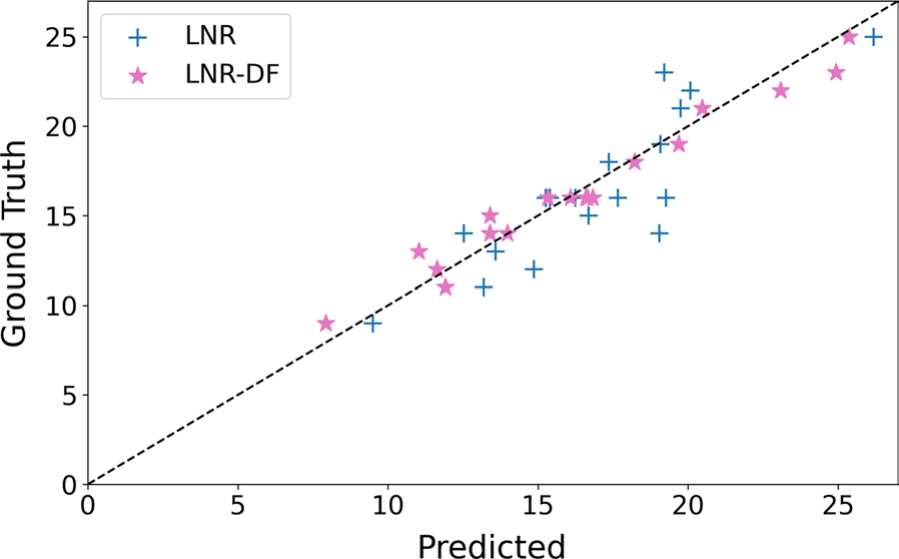
Scatter chart of LNR and LNR-DF with two-stage feature selection

Meanwhile, we draw a scatter plot (Fig. 3) of LNR and LNR-DF with the two-stage feature selection, where the x-axis and y-axis refer to predicted and ground truth HAMD-17 score, respectively. As shown in Fig. 3, most of the scatter points are nearby the diagonal line which means the presented two-stage feature selection method improves the MDD severity assessment performance very well, particularly with the combination of the derived $\beta /\alpha$ features.

### Hyperparameters tuning

**Fig. 4:**
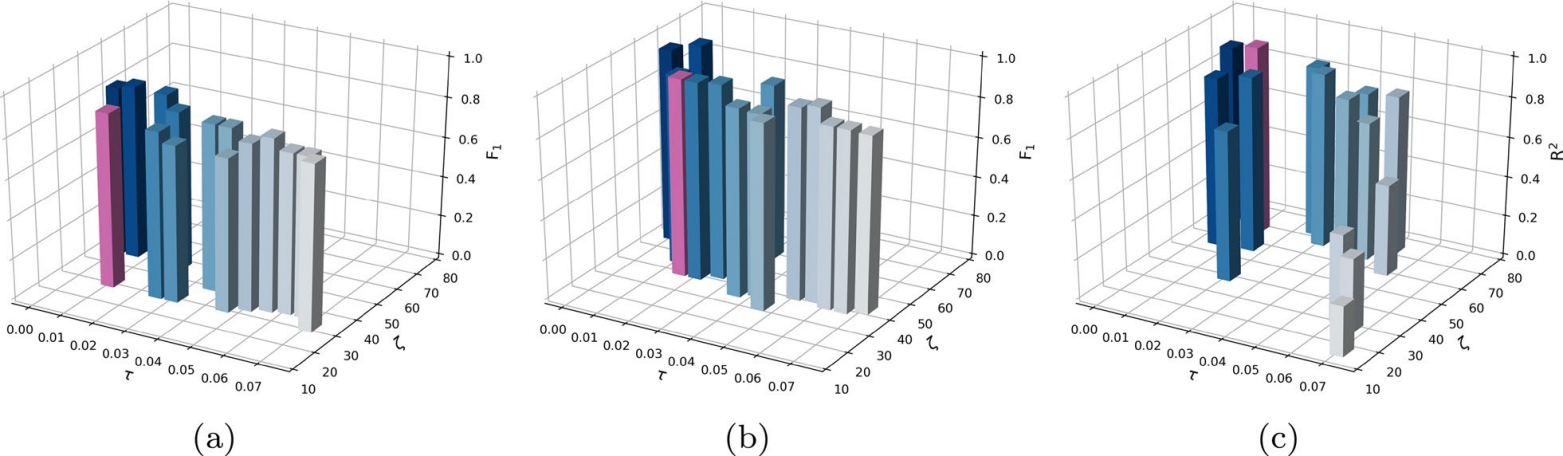
The process of determining $\tau$ and $\zeta$ according to LR, SVM, and LNR respectively in feature selection: **a** LR, **b** SVM, **c** LNR

In this section, the tuning of two key hyperparameters of the proposed framework is introduced briefly, where the two hyperparameters of the proposed framework are the threshold value $\tau$ for PCC feature selection and the number of features $\zeta$ selected by RFE. To obtain the optimal hyperparameters, the grid search technique is utilized for searching the optimal combination of $\tau$ and $\zeta$. Specifically, the hyperparameter $\tau$ is tuning from 0 .01 to 0.07 with a step of 0.01 while the hyperparameter $\zeta$ from 192 to 1 with a step of 1. As shown in Fig. 4, take LR-DF, SVM-DF, and LNR-DF for example, and it is noted that the hyperparameter $\tau$ is 0.01 for LR-DF and LNR-DF, and 0.015 for SVM-DF, while the hyperparameter $\zeta$ is different among them. The optimal hyperparameters $\zeta$ are 26, 36, and 63 in terms of LR-DF, SVM-DF, and LNR-DF, respectively, when they achieved the best performance. Furthermore, the key hyperparameters of $\tau$ and $\zeta$ of all MDD detection models and MDD severity assessment models are listed in Table 4 used in this work.

**Table 4:** Hyper-parameter of feature selection

Feature selection	Method	$\tau$	$\zeta$	*F*_1_	*R*^2^
–	SVM	–	154	0.4510	–
	SVM-DF	–	192	0.5144	–
	LNR	–	–	–	–
	LNR-DF	–	–	–	–
PCC	SVM	0.055	–	0.5306	–
	SVM-DF	0.055	–	0.5766	–
	LNR	0.210	–	–	0.0644
	LNR-DF	0.185	–	–	0.0927
RFE	SVM	–	43	0.9385	–
	SVM-DF	–	53	0.9667	–
	LNR	–	53	–	0.8474
	LNR-DF	–	66	–	0.8962
PCC and RFE	SVM	0.040	38	0.9581	–
	SVM-DF	0.015	36	0.9846	–
	LNR	0.010	59	–	0.8812
	LNR-DF	0.010	63	–	0.9479

### Statistical analysis

In order to more reasonably explain the significance of the $\beta /\alpha$ features and two-stage feature selection to model improvement, we repeat the experiment 5 times and conduct statistical power analysis in MDD detection and MDD severity assessment by calculating the *P* value between the method with the best performance and other methods in our framework respectively. Here, we leverage three levels to mark the significance (*,**,***), which represent the *P* value greater than or equal to 0.05, the *P* value between 0.05 and 0.01, and the *P* less than 0.01. As shown in Table 5, it is demonstrated that the SVM-DF with two-stage feature selection is superior to the compared methods significantly, especially in $F_{1}$ score, where *P* value between methods are much less than 0.01. Similarly, Table 6 shows that the LNR-DF with two-stage feature selection is superior to other methods that do not employ $\beta /\alpha$ features and two-stage feature selection simultaneously. The statistical analysis results mean that the $\beta /\alpha$ features and two-stage feature selection employed in our framework are effective and necessary.

**Table 5:** Statistical power analysis between SVM-DF with two-stage feature selection and other MDD detection methods in the framework

Feature selection	Model	*P* value				
		*Sec*	*Prec*	*Spec*	*Acc*	*F*_1_
–	LR	1.47e−05***	9.73e−06***	7.91e−06***	1.58e−06***	9.40e−06***
	SVM	1.52e−05***	7.80e−06***	1.67e−04***	6.50e−06***	2.80e−06***
	LR-DF	8.90e−07***	6.39e−06***	2.43e−05***	3.95e−06***	4.27e−06***
	SVM-DF	2.72e−04***	4.82e−05***	3.28e−05***	1.70e−06***	3.67e−05***
PCC	LR	4.81e−05***	7.46e−06***	2.64e−05***	1.53e−06***	3.66e−06***
	SVM	6.10e−06***	2.76e−06***	3.39e−04***	1.11e−05***	7.80e−07***
	LR-DF	8.37e−06***	1.44e−05***	1.90e−04***	5.78e−06***	1.30e−07***
	SVM-DF	4.61e−04***	2.99e−06***	2.30e−04***	2.44e−06***	1.26e−05***
RFE	LR	3.88e−04***	1.42e−03***	3.99e−04***	2.88e−04***	4.41e−04***
	SVM	8.64e−03***	2.59e−03***	4.07e−03***	2.55e−03***	2.61e−03***
	LR-DF	1.97e−03***	1.88e−02**	2.18e−02**	7.23e−05***	1.28e−04***
	SVM-DF	1.61e−02**	3.74e−01*	3.85e−01*	5.37e−02*	2.86e−02**
PCC and RFE	LR	1.44e−04***	5.34e−04***	1.78e−04***	5.68e−05***	1.21e−04***
	SVM	2.08e−01*	1.70e−01*	1.84e−01*	9.50e−03***	3.60e−03***
	LR-DF	3.16e−03***	4.47e−03***	3.99e−03***	7.74e−04***	9.18e−04***

*P ≥ 0.05; **0.05 > *P* ≥ 0.01; ***P < 0.01

**Table 6:** Statistical power analysis between LNR-DF with two-stage feature selection and other MDD severity assessment methods in the framework

Feature selection	Model	*P* value		
		*L*_*MAE*_	*L*_*MSE*_	*R*^2^
–	LNR	7.43e−06***	4.61e−05***	2.43e−04***
	LNR-DF	4.07e−06***	5.48e−05***	9.15e−05***
PCC	LNR	4.00e−08***	6.00e−08***	4.00e−08***
	LNR-DF	7.90e−07***	1.40e−07***	3.00e−08***
RFE	LNR	1.60e−02**	1.81e−02**	1.63e−02**
	LNR-DF	3.79e−03***	7.15e−03***	2.78e−03***
PCC and RFE	LNR	1.25e−02**	1.44e−02**	1.10e−02**

*P ≥ 0.05; **0.05 > *P* ≥ 0.01; ***P < 0.01

### Selected feature analysis

**Fig. 5:**
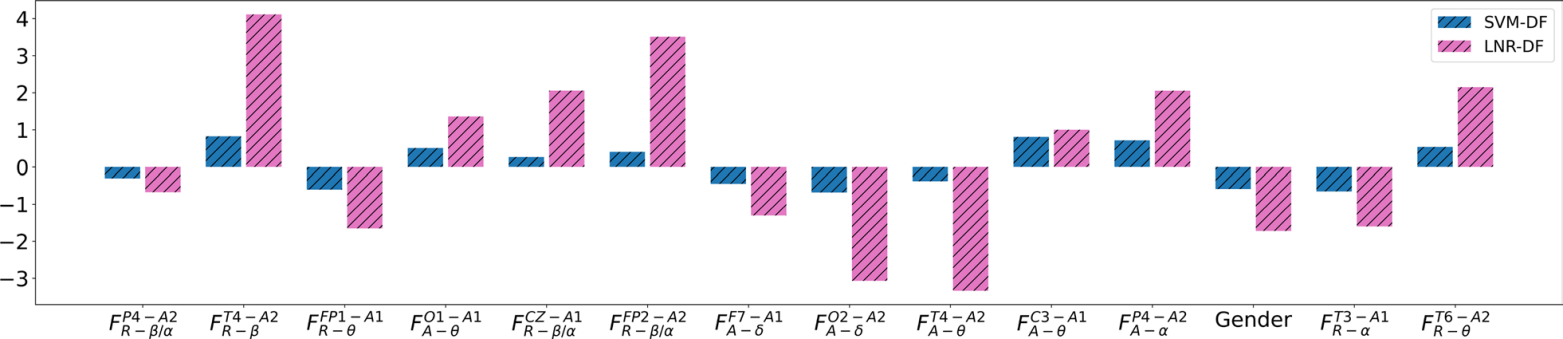
Weight importance of selected features shared by SVM-DF and LNR-DF

Figure 5 shows the shared selected features by MDD detection model SVM-DF and MDD severity assessment model LNR-DF. Obviously, we can see that the weight importance of selected features is quite different between SVM-DF and LNR-DF due to the difference in their principles for defining models. However, the direction of weight importance is identical between SVM-DF and LNR-DF, which means that the selected features have similar effects on the performance of MDD detection and MDD severity assessment. Meanwhile, it is observed that three derived $\beta /\alpha$ features of $F_{R-\beta /\alpha }^{P4-A2}$, ${F_{R-\beta /\alpha }^{CZ-A1}}$, and ${F_{R-\beta /\alpha }^{FP2-A2}}$ are selected by both MDD detection and MDD severity assessment models, which are more than 21 %. It further verifies our findings of derived features are quite effective in the models of MDD detection and severity assessment.

### Limitations

Since the size of the dataset used in this study is 92, our study is a pilot study, and we employ the SVM, LR, and LNR as the base model for MDD detection and MDD severity assessment, with the merits of small-size building models. To further conquer the over-fitting risk, the cross-validation technique is also utilized. Even so, it still potentially has the over-fitting risk for MDD detection and MDD severity assessment models.

On the other hand, the original EEG signals cannot be obtained from the commercial EEG device named Nerron-spectrum-5 as the data format for medical devices is usually commercial confidence. Herewith, the features used in this study are extracted by the EEG device directly. The noise would be introduced into extracted features due to the uncertainty of EEG signals quality, which would reduce the generality of the proposed framework potentially.

## Conclusions

In this study, we propose an automatic EEG-based MDD detection framework. In the proposed framework, the high MDD correlation features named $\beta /\alpha$ features are derived; a two-stage feature selection method is employed for well-selected correlated features to improve the model performance; three sample-sized machine learning methods of LR, SVM, and LNR as base models are utilized for MDD detection and MDD severity assessment. Experiment results show that the proposed framework achieves an optimal $F_{1}$ score of 0.9846 in MDD detection, and achieves an optimal $R^2$ of 0.9479 in MDD severity assessment. Meanwhile, the findings of the derived EEG-based $\beta /\alpha$ features can greatly improve the performance of MDD detection and MDD severity assessment. For MDD detection, the derived features would improve the $F_{1}$ score by over 2 % and 11 % in terms of SVM and LR, respectively. As for MDD severity assessment, the derived features would improve the $R^{2}$ of LNR from 0.8812 to 0.9479. Meanwhile, the base models used in this study are LR, SVM with linear kernel, and LNR, all of which are linear and with the merits of small sample size and interpretability. It means that the proposed framework can be potentially deployed into a medical system to provide MDD detection services to help physicians to screen out MDD patients.

Concerning the sample size of our experimental data is not big enough, in future work, we are intended to continue collecting more samples to expand our dataset and time retrain the model in the case of data added incrementally [50], which will better improve the generalization of our framework. In addition, more samples can be generated using sophisticated augmentation methods like Generative Adversarial Networks (GANs) [51]. Furthermore, we will consider using interpretable deep learning models [52] to improve the interpretability of our framework, to better assist clinical diagnosis.

## Data Availability

The data that support the findings of this study are available from Shenzhen Traditional Chinese Medicine Hospital but restrictions apply to the availability of these data, which were used under license for the current study, and so are not publicly available. Data are however available from the author named Xingxian Huang upon reasonable request and with permission of Shenzhen Traditional Chinese Medicine Hospital.
